# Performance evaluation of cetacean species distribution models developed using generalized additive models and boosted regression trees

**DOI:** 10.1002/ece3.6316

**Published:** 2020-05-11

**Authors:** Elizabeth A. Becker, James V. Carretta, Karin A. Forney, Jay Barlow, Stephanie Brodie, Ryan Hoopes, Michael G. Jacox, Sara M. Maxwell, Jessica V. Redfern, Nicholas B. Sisson, Heather Welch, Elliott L. Hazen

**Affiliations:** ^1^ National Marine Fisheries Service National Oceanic and Atmospheric Administration Ocean Associates, Inc., Under Contract to Southwest Fisheries Science Center La Jolla CA USA; ^2^ Institute of Marine Science University of California Santa Cruz Santa Cruz CA USA; ^3^ ManTech International Corporation Solana Beach CA USA; ^4^ Marine Mammal and Turtle Division Southwest Fisheries Science Center National Marine Fisheries Service National Oceanic and Atmospheric Administration La Jolla CA USA; ^5^ Marine Mammal and Turtle Division Southwest Fisheries Science Center National Marine Fisheries Service National Oceanic and Atmospheric Administration Moss Landing CA USA; ^6^ Moss Landing Marine Laboratories San Jose State University Moss Landing CA USA; ^7^ Environmental Research Division Southwest Fisheries Science Center Monterey CA USA; ^8^ Physical Sciences Division Earth System Research Laboratory Boulder CO USA; ^9^ School of Interdisciplinary Arts and Sciences University of Washington Bothell WA USA; ^10^ Anderson Cabot Center for Ocean Life New England Aquarium Boston MA USA; ^11^ Department of Biological Sciences Old Dominion University Norfolk VA USA

**Keywords:** boosted regression tree, California Current, cetacean, generalized additive model, habitat model, species distribution model

## Abstract

Species distribution models (SDMs) are important management tools for highly mobile marine species because they provide spatially and temporally explicit information on animal distribution. Two prevalent modeling frameworks used to develop SDMs for marine species are generalized additive models (GAMs) and boosted regression trees (BRTs), but comparative studies have rarely been conducted; most rely on presence‐only data; and few have explored how features such as species distribution characteristics affect model performance. Since the majority of marine species BRTs have been used to predict habitat suitability, we first compared BRTs to GAMs that used presence/absence as the response variable. We then compared results from these habitat suitability models to GAMs that predict species density (animals per km^2^) because density models built with a subset of the data used here have previously received extensive validation. We compared both the explanatory power (i.e., model goodness of fit) and predictive power (i.e., performance on a novel dataset) of the GAMs and BRTs for a taxonomically diverse suite of cetacean species using a robust set of systematic survey data (1991–2014) within the California Current Ecosystem. Both BRTs and GAMs were successful at describing overall distribution patterns throughout the study area for the majority of species considered, but when predicting on novel data, the density GAMs exhibited substantially greater predictive power than both the presence/absence GAMs and BRTs, likely due to both the different response variables and fitting algorithms. Our results provide an improved understanding of some of the strengths and limitations of models developed using these two methods. These results can be used by modelers developing SDMs and resource managers tasked with the spatial management of marine species to determine the best modeling technique for their question of interest.

## Graphical abstract

Species distribution models (SDMs) were developed for a taxonomically diverse suite of cetaceans using a robust set of systematic survey data within the California Current Ecosystem. Both boosted regression tress and generalized additive models were developed and their explanatory and predictive performance compared in light of features such as species distribution characteristics. Results have direct relevance for understanding the accuracy of SDMs and associated implications for marine management and conservation.

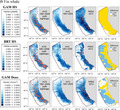

## 1 INTRODUCTION

Species distribution models (SDMs) are widely recognized as important marine spatial planning tools because they can describe and predict the distribution patterns of highly mobile marine species. SDMs have been developed for a wide range of marine predators and used to establish marine conservation areas, guide fisheries management, and assess risks posed by anthropogenic activities (Abrahms et al., 2019; Benson et al., 2011; Gilles et al., 2016; Hartog, Hobday, Matear, & Feng, 2011; Hazen et al., 2017; Hobday, Hartog, Timmis, & Fielding, 2010; Keller, Garrison, Baumstark, Ward‐Geiger, & Hines, 2012; Louzao et al., 2006; Redfern et al., 2019; Welch, Brodie, Jacox, Bograd, & Hazen, 2019). A variety of modeling techniques have been used to develop SDMs, including generalized additive models (GAMs), generalized linear models (GLMs), boosted regression trees (BRTs), Random Forests (RFs), and maximum entropy (MaxEnt) models (Austin, 2007; Elith et al., 2006; Hegel, Cushman, Evans, & Huettmann, 2010; Mi, Huettmann, Guo, Han, & Wen, 2017; Oppel et al., 2012; Robinson, Nelson, Costello, Sutherland, & Lundquist, 2017; Shabani, Kumar, & Ahmadi, 2016).

There is an extensive body of literature confirming the predictive ability of GAMs for cetacean ecological data (e.g., Becker et al., 2012, 2014, 2018; Best et al., 2012; Cañadas & Hammond, 2008; Ferguson, Barlow, Fiedler, Reilly, & Gerrodette, 2006; Gilles, Adler, Kaschner, Scheidat, & Siebert, 2011; Hedley, Buckland, & Borchers, 1999; Keller et al., 2012; Lambert, Mannocci, Lehodey, & Ridoux, 2014; Mannocci et al., 2014). Recently, there has been increased interest in machine‐learning techniques such as BRTs (Elith, Leathwick, & Hastie, 2008) and RFs (Breiman, 2001) that are able to test and fit multiple interactions among predictors and are tolerant of outliers, collinearity, and irrelevant predictors, making these techniques powerful for analyzing complex ecological relationships (Breiman, 2001; De'Ath, 2007; Elith & Leathwick, 2009; Elith et al., 2008; Leathwick, Elith, Francis, Hastie, & Taylor, 2006).

When evaluating the performance of SDMs, there has been an emphasis on statistically comparing models with different conceptual frameworks (Austin, 2007; Elith & Leathwick, 2009; Franklin, 1998; Guisan & Thuiller, 2005). A robust comparison sometimes involves simulated data so that the “true” relationship between the response and predictor variables is known and results are not confounded by differences in responses, predictors, or model parameterization (Austin, 2007; Brodie et al., 2019). The use of real data is also valuable, because cross‐validation with spatially and/or temporally novel datasets can be used to quantitatively assess model performance with data that were not used to build the models (Hijmans, 2012; Shabani et al., 2016). Comparing the results from different models built with real data can provide important insights for the spatial management of marine species (Robinson et al., 2011) and increase our understanding of both the strengths and weaknesses of different modeling techniques, helping to guide future modeling efforts.

The majority of SDM comparison studies using real data have focused on terrestrial species (e.g., Elith et al., 2006; Franklin, Wejnert, Hathaway, Rochester, & Fisher, 2009; Robinson et al., 2011; Segurado & Araújo, 2004; Shabani et al., 2016; Syphard & Franklin, 2009). Comparative modeling studies have been developed for marine species such as fish (BRTs, RFs, GAMS; Leathwick et al., 2006; Stock et al., 2019), seabirds (GLMs, GAMs, RFs, BRTs, and MAXENT; Oppel et al., 2012), and additional taxa (Robinson et al., 2017), but results from these marine‐based model comparisons have not been consistent across species. Few comparison studies have focused on cetacean SDMs (e.g., GLMs vs. GAMs, Becker et al., 2010; GAMs vs. MAXENT, Fiedler et al., 2018). Studies that have compared cetacean SDMs have primarily used nonsystematic survey data for model development (e.g., GLMs, GAMs, BRTs, MAXENT, and support vector machines; Derville, Torres, Iovan, & Garrigue, 2018; BRTs vs. generalized additive mixed models [GAMMs], Abrahms et al., 2019, Hazen et al., 2017) and have rarely explored how species distribution characteristics (i.e., spatial distribution and habitat preference) affect model performance. Finally, ensemble modeling has emerged as a robust method for combining multiple modeling results (e.g., Abrahms et al., 2019; Forney, Becker, Foley, Barlow, & Oleson, 2015; Marmion, Parviainen, Luoto, Heikkinen, & Thuiller, 2009; Oppel et al., 2012; Pikesley et al., 2013; Scales et al., 2016; Woodman et al., 2019), prompting a need to better understand the strengths and weaknesses of different modeling approaches to inform uncertainty‐based weightings.

U.S. west coast waters are habitat for over 25 cetacean species, which are all protected under the Marine Mammal Protection Act (MMPA), and some species are also protected under the Endangered Species Act (ESA). Given the overlap of cetacean habitat with hotspots of human use such as the shipping lanes leading into the ports of San Francisco and Long Beach (Moore et al., 2018), there is a need to understand the spatial and temporal habitat use of these species. SDMs for cetaceans have been developed for U.S. west coast waters from systematic ship survey data collected by the Southwest Fisheries Science Center (SWFSC) since 1991, and these GAMs have been extensively evaluated using cross‐validation (Barlow et al., 2009; Becker et al., 2010; Forney, 2000; Forney et al., 2012) and predictions on independent datasets (Barlow et al., 2009; Becker et al., 2012, 2014, 2018; Calambokidis et al., 2015; Forney et al., 2012). The most recent models provide spatially explicit density predictions at a 0.1˚ (approximately 10km x 10km) grid resolution (Becker et al., 2016), and they have been used by the Navy to assess potential impacts on cetaceans as required by U.S. regulations such as the MMPA and ESA (U.S. Department of the Navy, 2013, 2015, 2017). However, a comparison between different model types developed using these systematic data has not been performed, despite the potential for insight into both model performance and management of these species.

**FIGURE 1:**
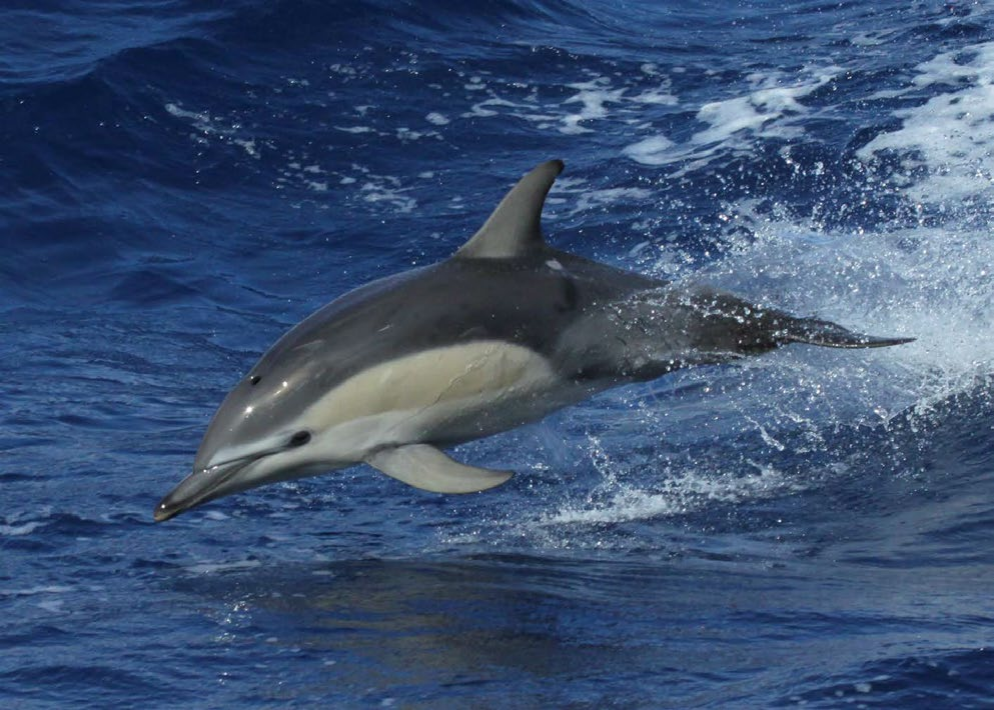
Short‐beaked common dolphin (*Delphinus delphis delphis*) in the California Current Ecosystem study area. Photograph taken by K.A. Forney under NMFS Permit No. 19091

The objective of this study was to compare the explanatory and predictive power of the two most prevalent modeling frameworks used to develop SDMs for cetaceans, BRTs and GAMs, and evaluate how species distribution characteristics affect model performance. Ultimately, a better understanding of model performance will improve the application of SDMs for marine spatial planning and conservation efforts. We used systematic cetacean survey data collected by SWFSC between 1991 and 2014 to develop SDMs for seven taxonomically diverse species or subspecies that have different spatial distributions and habitat preferences: short‐beaked common dolphin (*Delphinus delphis delphis*; Figure 1), long‐beaked common dolphin (*Delphinus delphis bairdii*), striped dolphin (*Stenella coeruleoalba*), northern right whale dolphin (*Lissodelphis borealis*), Risso's dolphin (*Grampus griseus*), fin whale (*Balaenoptera physalus*), and humpback whale (*Megaptera novaeangliae*). We selected these species to provide a comparison of (a) species with widespread distributions (short‐beaked common dolphin and fin whale) versus restricted distributions (long‐beaked common dolphin and humpback whale) in the study area, (b) species that occur in more dynamic nearshore habitat (northern right whale dolphin) versus less variable offshore habitat (striped dolphin), and (c) a species for which previous density GAMs did not perform as well as expected (Risso's dolphin; e.g., Becker et al., 2010; Forney et al., 2012). Since the majority of BRTs developed for marine species have been used to predict habitat suitability (probability of presence or relative abundance), which is then converted to presence/absence, ideally using a meaningful threshold value (Abrahms et al., 2019; Brodie et al., 2018; Derville et al., 2018; Hazen et al., 2017; Maxwell et al., 2019; Scales et al., 2017), we first compared BRTs to GAMs that used presence/absence as the response variable. We then compared results from these models to GAMs that predict absolute density (animals per km^2^), since density models built with these data have previously received extensive validation. Results enhance our scientific understanding of how the ecology and life history of different species affect the accuracy of models developed in different frameworks and, thus, the accuracy of potential management advice.

## 2 METHODS

### 2.1 Survey data

**FIGURE 2:**
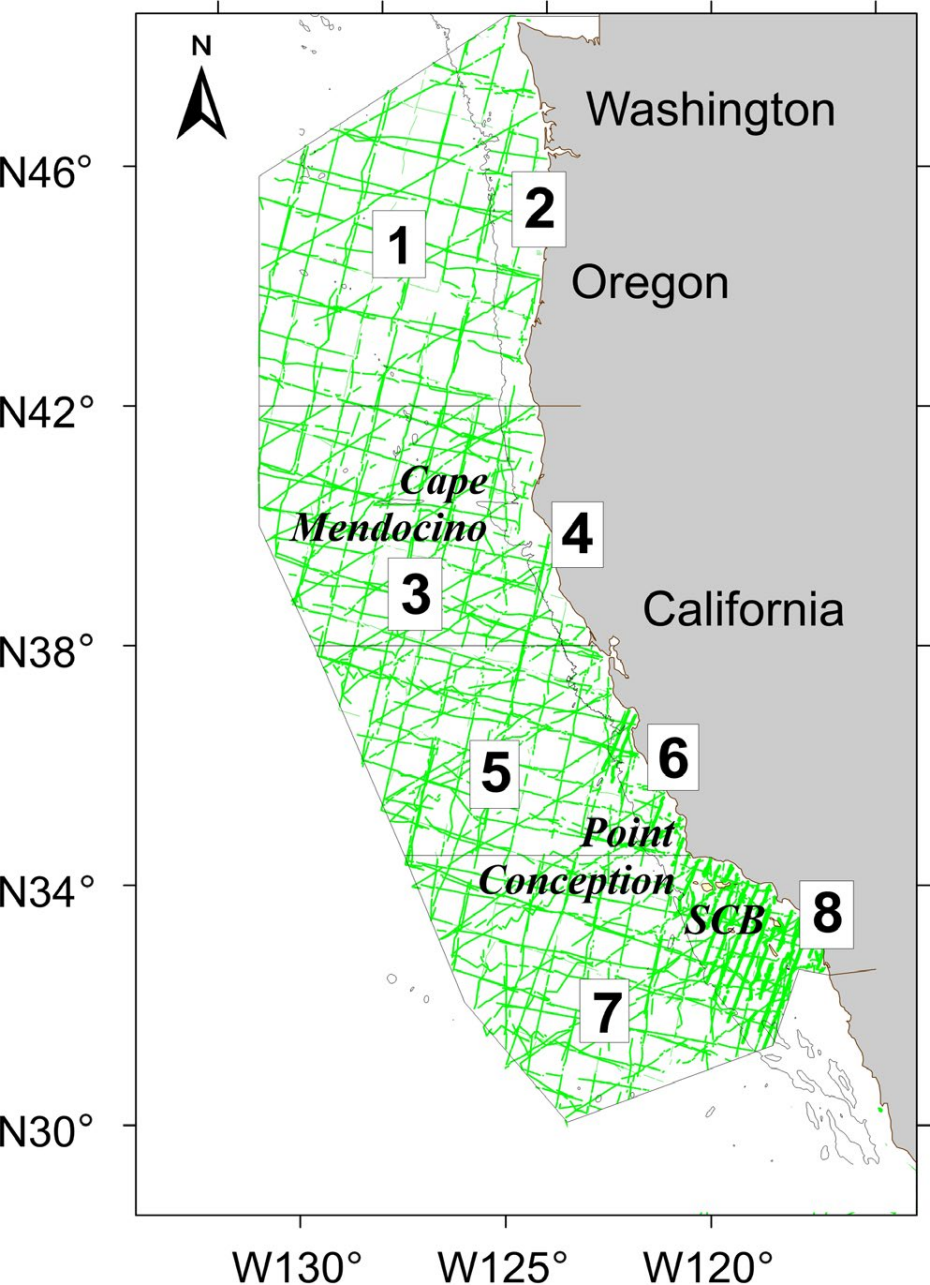
Completed transects for the Southwest Fisheries Science Center systematic ship surveys conducted between 1991 and 2014 in the California Current Ecosystem study area and the eight geographic strata used to evaluate the accuracy of the spatial patterns of predicted habitat suitability/density. The four north–south strata are consistent with those used for line‐transect abundance estimation (Barlow & Forney, 2007) and an offshore–onshore division occurs at the 2,000 m isobath. Region names are as follows: (1) OR/WA west, (2) OR/WA east, (3) NorCA west, (4) NorCA east, (5) CenCA west, (6) CenCA east, (7) SoCA west, and (8) SoCA east. The green lines show on‐effort transect coverage in Beaufort sea states ≤ 5. Also shown are names of geographic places mentioned in the text (SCB, Southern California Bight)

Cetacean survey data used to build the SDMs were collected in the California Current Ecosystem (CCE) during the summer and fall (July through early December) of 1991, 1993, 1996, 2001, 2005, 2008, 2009, and 2014 using systematic line‐transect methods (Buckland et al., 2001). With the exception of 2009, which covered a limited area to target common dolphins (Carretta, Chivers, & Perryman, 2011), transect lines were arranged in a systematic grid to provide even coverage of the survey region over the course of each survey. When combined across years, the surveys provided dense coverage of waters from the west coast of the United States to approximately 556 km offshore (Figure 2; Barlow, 2016; Barlow & Forney, 2007; Carretta et al., 2011). We used on‐effort sampling data from transect segments where Beaufort sea state (a wind index inversely correlated with animal detection rate) was ≤5. The survey protocol was the same for all years (see Barlow & Forney, 2007; Kinzey, Olson, & Gerrodette, 2000). Research vessels traveled at approximately 18 km/hr along predetermined transect lines while two experienced observers searched with pedestal‐mounted 25 × 150 binoculars (approximately 10–15 m above sea‐level depending on the research vessel). A third observer searched with unaided eyes and 7× hand‐held binoculars and also recorded data on survey conditions and cetacean sightings. When cetaceans were sighted, the research vessel approached the group as needed to identify the species and estimate the number of individuals in the group. All observers independently provided best, high, and low group size estimates; we averaged the best estimates for each species to obtain a single group size estimate for each sighting.

The modeling dataset was created by dividing continuous portions of survey effort into approximate 5‐km segments using the approach described by Becker et al. (2010). Species‐specific sighting data were assigned to each segment (total number of sightings and average group size), and habitat covariate values were derived based on the segment's geographic midpoint. Sighting data were truncated at a distance of 5.5 km perpendicular to the track line to eliminate the most distant groups (Buckland et al., 2001) and to maintain consistency with the species‐specific effective‐strip‐width estimates (key parameters in line‐transect density analyses that provide measures of how far animals are seen from the transect line) derived by Barlow, Ballance and Forney (2011) and used in this study to estimate cetacean densities.

### 2.2 Habitat variables

As is the case for most cetacean SDMs, the selected habitat predictors are most likely proxies for unmeasured underlying ecological processes driving species distributions. The same suite of predictor variables was used for both model types (GAM, BRT) and included a combination of dynamic, bathymetric, and spatial covariates as described below. We also offered year as a potential predictor in all models to capture population trends for species whose abundance has increased substantially during the time period considered in our analyses: the short‐beaked common dolphin (Barlow, 2016), fin whale (Moore & Barlow, 2011), and humpback whale (Barlow, Calambokidis, et al., 2011).

#### 2.2.1 Dynamic variables

Dynamic variables used in this study are defined as those that change on temporal scales of days to weeks in the CCE study area (Bograd et al., 2009). Dynamic predictors derived from a data assimilative CCE configuration of the Regional Ocean Modeling System (ROMS), produced by the U.C. Santa Cruz Ocean Modeling and Data Assimilation group (Moore et al., 2011; Neveu et al., 2016), have been shown to be effective in similar SDMs for these species in this study area (Becker et al., 2016, 2017, 2018). We used daily output for each ROMS variable at the 0.1 degree (~10 km) horizontal resolution of the model. We used output from both a historical reanalysis (1980–2010; Neveu et al., 2016) and a near‐real‐time data assimilation system (2011–present; Moore et al., 2013; oceanmodeling.ucsc.edu) to cover the broad temporal span of our survey data (1991–2014). Both systems provide data‐constrained state estimates for our study area, but they differ in assimilation details and the specific data used. We limited the predictors to those consistent between the two sources (Becker et al., 2017): sea surface temperature (SST) and its standard deviation (sdSST), calculated for a 3 × 3 pixel box centered on the pixel containing the modeling segment midpoint, mixed layer depth (MLD, defined by a 0.5°C deviation from the SST), sea surface height (SSH), and its standard deviation, sdSSH. An offset (+0.035 m) was applied to the near‐real‐time SSH data to match the historical reanalysis dataset, which had a different reference level (Scales et al., 2017).

#### 2.2.2 Bathymetric variables

Bathymetric data were derived from ETOPO1 (obtained from https://www.ngdc.noaa.gov/mgg/global/global.html; 0.1‐degree resolution; Amante & Eakins, 2009). Given its prevalence as an important predictor in past modeling studies in this ecosystem (e.g., Becker et al., 2010, 2016, 2018), we selected water depth (m) as a habitat variable to represent bathymetry, obtained for the midpoint of each transect segment.

#### 2.2.3 Spatial variables

Latitude and longitude are prevalent as covariates in many cetacean modeling studies (e.g., Cañadas & Hammond, 2008; Forney et al., 2015; Hedley et al., 1999; Pirotta, Matthiopoulos, MacKenzie, Scott‐Hayward, & Rendell, 2011; Tynan et al., 2005; Williams, Hedley, & Hammond, 2006). They were included as covariates in our study as they have been shown to increase the explanatory power of SDMs because they often account for unmeasured variables that might be important for driving species distributions (Becker et al., 2018). The inclusion of spatial covariates prohibits predictions outside of the study area, but allowed us to explicitly evaluate how the different modeling methods handled discrete spatial data.

#### 2.2.4 Interaction terms

One of the advantages of BRTs is their ability to automatically fit interactions between predictor variables, while interactions must be explicitly defined when fitting GAMs. Previous comparative modeling studies have recognized the importance of interaction terms in SDMs and explicitly included them in GAMs to enable a more equitable comparison (Leathwick et al., 2006). Given the importance of spatial interaction terms in past cetacean SDMs (Becker et al., 2016, 2018; Forney, 2000; Palacios et al., 2019; Yuan et al., 2017), bivariate interaction terms between latitude and each of the dynamic variables (SST, MLD, and SSH) were included individually when building the GAMs (see below for a description of the GAM modeling framework).

### 2.3 Generalized additive models

Both the habitat suitability and density GAMs were developed in R (R Core Team, 2017) using the package “mgcv” (Wood, 2011), which uses cross‐validation as part of the model selection process. We used restricted maximum likelihood (REML) to optimize the parameter estimates and a variable selection process that uses a shrinkage approach to modify the smoothing penalty and effectively remove nonsignificant variables from the model (Marra & Wood, 2011). REML provides more accurate smooth term estimates than other methods such as Akaike's information criterion (AIC) that have been shown to be prone to undersmoothing (Marra & Wood, 2011). To ensure that models were not overfit, we also removed variables that had *p*‐values > .05 and then refit the models to ensure that all remaining variables had *p*‐values < .05 (Redfern et al., 2017; Roberts et al., 2016). Pairwise interaction terms were considered separately in the GAMs to avoid overfitting and to aid in the ecological interpretation of the interaction term (Becker et al., 2016). Correlations among the predictor variables in our study ranged from 0.003 to 0.66 (absolute values), but mgcv is robust to such effects (termed “concurvity”; Wood, 2008).

#### 2.3.1 Habitat suitability GAMs

To develop presence/absence models from the systematically collected survey data, we assigned values of 1 to those segments that included sightings and values of 0 to those segments with no sightings. We fit binomial GAMs using a logit link function so that the resultant models describe the probability of species presence, also termed “habitat suitability” (Brodie et al., 2018) or “habitat preference” (Hazen et al., 2017).

#### 2.3.2 Density GAMs

The methods used to develop the density GAMs followed those described in Becker et al. (2018). For species that occur in small groups (i.e., fin and humpback whales), we fit a single response model using the number of individuals per transect segment as the response variable with a Tweedie distribution to account for overdispersion (Miller, Burt, Rexstad, Thomas, & Gimenez, 2013). The other species are all members of the Family Delphinidae that tend to occur in groups with large and variable sizes, so we fit separate encounter rate and group size models. Encounter rate (number of sightings per segment) models were fit with all transect segments using a Tweedie distribution (i.e., assume the number of groups sighted per segment is Tweedie distributed, e.g., Foster & Bravington, 2013). Group size models were fit with only those transect segments that included sightings, using the natural log of group size as the response variable and a Gaussian link function. To account for observed geographic differences in the size of delphinid groups (Barlow, 2015; Cañadas & Hammond, 2008; Ferguson et al., 2006), group size was modeled using a tensor product smooth of latitude and longitude (Becker et al., 2018; Wood, 2003). The natural log of the effective area searched (described below) was included as an offset in both the single response and encounter rate models to account for both varying segment lengths and the different detection probabilities recorded during the surveys.

Density (number of animals per km^2^) was estimated by incorporating the model results into the standard line‐transect equation (Buckland et al., 2001):

(1) $$D_{i}=\frac{n_{i}\cdot s_{i}}{A_{i}}$$

where *i* is the segment, *n* is the number of sightings, *s* is the average group size, and *A* is the effective area searched:

(2) $$A_{i}=2\cdot L_{i}\cdot {\text{ESW}}_{i}\cdot g{\left(0\right)}_{i}$$

where *L* is the length of the effort segment (km), ESW is the effective strip half‐width (km), and *g*(0) is the probability of detection on the transect line. Following the methods of Becker et al. (2016), species‐specific estimates of ESW and *g*(0) were derived based on the recorded detection conditions on each modeling segment using coefficients estimated by Barlow, Ballance, et al. (2011) for ESW and Barlow (2015) for *g*(0).

### 2.4 Boosted regression trees

BRTs use machine‐learning methods whereby predictions from single‐tree models are combined to maximize predictive performance (Elith et al., 2008). We fit the BRTs in R (R Core Team, 2017) using the package “dismo” (Elith et al., 2008), following the methods described in Leathwick et al. (2006) and Elith et al. (2008). For each set of models, we built presence–absence BRTs specifying a binomial distribution consistent with the habitat suitability GAMs described above. The BRTs were assigned a tree complexity of 3, a bag fraction of 0.6, and we adjusted the learning rate (“shrinkage”) for each model to ensure that at least 1,000 trees were included in the final model configuration (Elith et al., 2008). Due to the tendency of BRTs to overfit (Leathwick et al., 2006), we included a random number as a potential predictor in each model run to compare against the other variables’ contributions; only relevant predictors (i.e., those more significant than the random variable) were included in the final BRTs (Eguchi, Benson, Foley, & Forney, 2017).

We built BRTs for each species using four combinations of variables to reduce the potential for overfitting and to explore the effect of including geographic (latitude, longitude) terms in the models: (a) dynamic and bathymetric variables only; (b) dynamic, bathymetric, and latitude; (c) dynamic, bathymetric, and longitude; (d) all variables. These models were compared using explained deviance, the receiver operating characteristic curve (AUC; Fawcett, 2006), and the true skill statistic (TSS; Allouche, Tsoar, & Kadmon, 2006), all metrics commonly used to assess BRTs (Brodie et al., 2018; Elith et al., 2006; Franklin et al., 2009; Oppel et al., 2012; Scales et al., 2017). Each BRT iteration is stochastic, and although generally the key variables (i.e., those with the most influence) are consistent among individual models runs, we used the best BRT of 10 model iterations for this analysis.

### 2.5 Model predictions

For each species, the three models (suitability BRT, suitability GAM, and density GAM) were each used to make predictions on distinct daily composites of environmental conditions for all 1991–2014 survey days (*n* = 1,312) used to develop the models. We used the average of all composites to represent expected long‐term patterns in species distributions that account for the varying oceanographic conditions during the 1991–2014 summer/fall SWFSC cetacean surveys. Log‐normal 90% confidence intervals for the spatial predictions principally reflect temporal variability in population density/habitat suitability since this has been shown to contribute the greatest source of uncertainty in these models (Barlow et al., 2009; Becker et al., 2014; Boyd et al., 2018; Ferguson et al., 2006). The prediction grid was clipped to the boundaries of the approximate 1,141,800‐km^2^ study area to ensure that predictions were not extrapolated outside the region used for model development.

### 2.6 Performance evaluation

The explanatory power of the models was compared using a set of established SDM performance metrics including AUC, TSS, and visual inspection of predicted and observed distributions during the 1991–2014 summer/fall SWFSC cetacean surveys (Barlow et al., 2009; Becker et al., 2010, 2016; Forney et al., 2012; Oppel et al., 2012; Scales et al., 2016; Woodman et al., 2019). AUC and TSS measure the discriminatory ability of an SDM and can be calculated using any type of prediction value. To calculate TSS for the GAM density models, we used the sensitivity–specificity sum maximization approach (Liu, Berry, Dawson, & Pearson, 2005) to obtain thresholds for species presence. To assess the ability of the models to predict spatial distribution patterns, we used the presence/absence GAMs and BRTs to estimate habitat suitability and the density GAMs to estimate abundance specific to eight geographic strata within the CCE study area (Figure 2): four north–south strata consistent with those used for line‐transect abundance estimation (Barlow & Forney, 2007), and an offshore–onshore division at the 2,000‐m isobath, which roughly represents the transition from the continental slope to the continental rise. The four north–south strata included waters off Oregon and Washington (322,200 km^2^ north of 42°N), northern California (258,100 km^2^ south of 42°N and north of Point Reyes at 38°N), central California (243,000 km^2^ between Point Conception at 34.5°N and Point Reyes), and southern California (318,500 km^2^ south of Point Conception). Given the different response variables, we used a nonparametric Spearman rank correlation test to compare the models’ ranked predicted values across the eight geographic strata to those derived from the actual survey data (Becker et al., 2014).

To compare the models’ ability to predict on novel data, we also built the models without the 2014 survey data and then used each of these models to predict on the 2014 environmental conditions of the summer/fall SWFSC survey. We selected 2014 for this evaluation because during this time waters in the CCE became anomalously warm as an unprecedented marine heatwave spread over the area (Bond, Cronin, Freeland, & Mantua, 2015; Cavole et al., 2016; Di Lorenzo & Mantua, 2016; Leising et al., 2015), providing a unique opportunity for cross‐validation. A previous study (Becker et al., 2018) assessed the ability of the density GAMs to predict abundance and distribution during this novel year for some of the species considered here, and given their success, we wanted to compare novel predictions from the presence/absence GAMs and BRTs. Following the methods of Becker et al. (2018), models were built with different combinations of the habitat variables and the model with the highest predictive performance was carried forward to represent that model type. For each of the three models, we then computed overall study area ratios of observed‐to‐predicted values and inspected predicted 2014 distribution patterns as compared to the 2014 survey observations (Barlow et al., 2009; Becker et al., 2010, 2016, 2018; Forney et al., 2012; Redfern et al., 2013).

Results were examined in light of the species‐specific characteristics that could affect model performance including study area distribution patterns and habitat preferences.

## 3 RESULTS

### 3.1 Explanatory performance

The 1991–2014 SWFSC surveys provided 82,659 km of on‐effort data in Beaufort sea states ≤5 which were used to develop the three different SDMs. The number of sightings available for modeling varied between species, ranging from 115 for northern right whale dolphin to 906 for short‐beaked common dolphin (Table 1). Key predictor variables selected by the two different (habitat suitability and density) GAMs and BRT fitting algorithms (Table 1) were consistent with those found in previous studies that used subsets of the same survey data (Barlow et al., 2009; Becker et al., 2010, 2016, 2018; Forney et al., 2012; Redfern et al., 2013), although the BRTs included the greatest number of predictor variables.

**TABLE 1:** Summary of the final GAM and BRT habitat suitability (HS) and GAM density (Dens) models built with the 1991–2014 survey data and the number of sightings available for modeling (*n*)

Species	Predictor variables	Expl.Dev.	AUC	TSS	Obs:Pred
Short‐beaked common dolphin (*n* = 906)					
GAM HS	LON:LAT + year +SST + SSH +MLD	12.2	0.77	0.41	1.00
BRT HS	LAT, SST, depth, SSH, MLD, SSTsd, SSHsd	27.93	0.90	0.63	1.00
GAM Dens	LON:LAT + year +SST + SSH +MLD	3.77	0.69	0.28	0.99
Long‐beaked common dolphin (*n* = 131)					
GAM HS	LON:LAT + SSHsd	48.80	0.98	0.92	1.00
BRT HS	LAT, depth, SST, SSH, SSTsd, SSHsd	57.09	0.99	0.92	1.01
GAM Dens	LON:LAT + SST	51.00	0.97	0.88	1.00
Northern right whale dolphin (*n* = 115)					
GAM HS	SST + depth +LON:LAT + MLD +SSH	13.80	0.84	0.53	1.00
BRT HS	SST, MLD, SSTsd, depth, SSH, SSHsd	51.75	0.98	0.88	1.00
GAM Dens	SST:LAT + depth	13.10	0.80	0.45	1.02
Striped dolphin (*n* = 151)					
GAM HS	LON:LAT + SST +SSH	10.60	0.79	0.46	1.00
BRT HS	depth, SST, SSH, LAT, MLD, LON	20.36	0.90	0.63	1.00
GAM Dens	SST:LAT + depth	3.44	0.70	0.33	0.97
Risso's dolphin (*n* = 182)					
GAM HS	LON:LAT + MLD	12.30	0.78	0.44	0.99
BRT HS	depth, SST, MLD, SSTsd, SSH, SSHsd	31.03	0.91	0.62	0.99
GAM Dens	LON:LAT + SST	9.95	0.74	0.41	0.98
Fin whale (*n* = 441)					
GAM HS	LON:LAT + year +SSH + SST +depth + SSHsd	13.70	0.81	0.47	1.00
BRT HS	SSH, SST, MLD, depth, SSTsd, SSHsd	42.22	0.96	0.70	1.00
GAM Dens	LON:LAT + year +SSH + MLD +SST + depth	10.50	0.74	0.38	0.86
Humpback whale (*n* = 360)					
GAM HS	LON:LAT + SST +year + depth +MLD + SSHsd	37.40	0.94	0.77	0.99
BRT HS	SST, depth, MLD, SSTsd, SSHsd, SSH, year	51.83	0.96	0.79	1.00
GAM Dens	LON:LAT + SST +year + depth +MLD	51.30	0.93	0.72	0.96

Note

Variable abbreviations are as follows: depth, bathymetric depth; LAT, latitude; LON, longitude; MLD, mixed layer depth; SSH, sea surface height; SSHsd, standard deviation of SSH; SST, sea surface temperature; SSTsd, standard deviation of SST. The “LON:LAT” and “SST:LAT” terms in the GAMs indicate an interaction term. All models for short‐beaked common dolphin, fin whale, and humpback whale were also offered a year covariate to capture their change in abundance during the 1991–2014 survey years (see text for details). Variables are listed in the order of their importance in each model. Comparative explanatory performance metrics (i.e., model goodness of fit) included percentage of explained deviance (Exp.Dev.), the area under the receiver operating characteristic curve (AUC), the true skill statistic (TSS), and the ratio of observed:predicted habitat suitability/density for the study area (Obs:Pred).

Year was included as a continuous variable in the final habitat suitability and density GAMs and BRT for humpback whale, capturing the increasing population trend of this species during the time period considered in our analysis (Barlow, Calambokidis, et al., 2011; Calambokidis, Barlow, Flynn, Dobson, & Steiger, 2017). Year was also a key predictor variable in the short‐beaked common dolphin and fin whale habitat suitability and density GAMs, but was not significant in the BRTs for these species (i.e., year had lower relative variable contribution than the random variable).

The overall study area ratios of observed‐to‐predicted density/habitat suitability values were very similar for the GAMs and BRTs, yet the percentage of explained deviance, AUC, and TSS metrics were generally highest for the BRTs and lowest for the density GAMs (Table 1). The nonparametric rank correlations were significant for all species for both types of GAMs and the BRTs, although the density GAM exhibited better performance overall (Table 2). AUC, TSS, and the rank correlations all measure the discriminatory ability of an SDM (i.e., how well a model separates occupied from unoccupied sites; Vaughan & Ormerod, 2005); however, results from the rank correlations suggest that the density GAMs were better able to capture large‐scale spatial distribution patterns throughout the study area.

**TABLE 2:** Summary of the Spearman rank correlation coefficients (Rs) for geographic regions within the study area based on observed values (i.e., estimates from the 1991–2014 survey data) and predictions from the final GAM and BRT habitat suitability (HS) and GAM density (Dens) models

Species	Rs GAM HS	Rs BRT HS	Rs GAM Dens
Short‐beaked common dolphin	0.976	0.929	1.000*
Long‐beaked common dolphin	0.792	0.792	0.798*
Northern right whale dolphin	0.643	0.786*	0.738
Striped dolphin	0.952	0.976*	0.976*
Risso's dolphin	0.762	0.738	0.833*
Fin whale	0.929*	0.881	0.929*
Humpback whale	0.833	0.833	0.833

Note

Regions are shown graphically in Figure 2. The critical value at ≤0.05 (1‐tailed test) with 7 degrees of freedom = 0.643. Significant correlations were found for all model predictions but the model(s) that exhibited better performance are marked with an asterisk (*).

**FIGURE 3:**
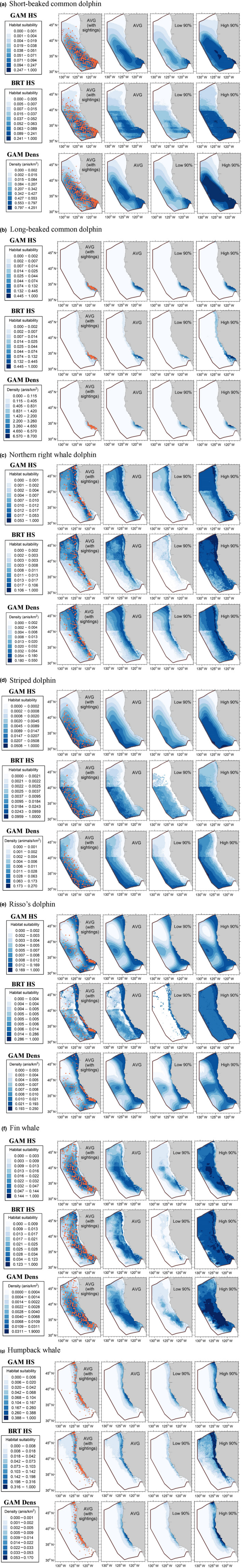
Predicted habitat suitability/density values and uncertainty measures from the 1991–2014 SDMs for (a) short‐beaked common dolphin, (b) long‐beaked common dolphin, (c) striped dolphin, (d), northern right whale dolphin, (e) Risso's dolphin, (f) fin whale, and (g) humpback whale. Panels show multiyear average (AVG) habitat suitability (HS)/density (Dens) based on daily predictions covering the survey periods (summer/fall 1991–2014), as well as the 90% confidence limits (low 90% and high 90%). To enable comparisons among the different model types, habitat probability/density values are presented in eight equal‐numbered bins based on the range of values for the 1991–2014 average, with a 9th color added to emphasize higher values within the upper 90% confidence limit. Predictions are shown for the study area (1,141,800‐km^2^). Orange dots in the average plots show actual sighting locations from the SWFSC summer/fall ship surveys for the respective species

The 1991–2014 daily composite average density/habitat suitability plots for the two model types showed similar overall distribution patterns for the majority of species, although there were obvious dissimilarities in portions of the study area (Figure 3). For example, the BRT for short‐beaked common dolphin showed an abrupt transition in habitat suitability at approximately 40°N associated with the Mendocino Escarpment, while both the GAMs showed a gradual decrease in density moving north of this latitude line (Figure 3a). Both types of GAM and the BRT predictions for fin whale showed this species’ widespread distribution throughout the study area, with areas of high density/habitat suitability extending from the Southern California Bight north to approximately 44°N, with areas of low density/habitat suitability in the southwestern portion of the study area (Figure 3f). North of 37°N the models differed, as the density GAM showed gradually lower density along the shelf where there were fewer sightings while the presence/absence GAM and BRT showed an abrupt lack of habitat suitability on the shelf extending continuously north to the U.S./Canadian border. All three models for humpback whale revealed a largely nearshore distribution, with highest density/habitat suitability extending from the northern portion of the Southern California Bight north to the U.S./Canadian border (Figure 3g); however, the BRT showed low‐to‐moderate habitat suitability in areas well offshore in the northern portions of the study area where there were no sightings of this species, despite good survey coverage (Figure 2).

Study area distribution patterns predicted by the three models were most similar for northern right whale dolphin (Figure 3c) and striped dolphin (Figure 3d) and differed most for long‐beaked common dolphin (Figure 3b) and Risso's dolphin (Figure 3e), most notably for the BRTs. Both types of GAM and the BRT showed a swath of high density/habitat suitability for Risso's dolphin extending along the coast of the entire study area. However, offshore regions of predicted high density/habitat suitability differed in extent. Two well‐defined offshore regions were predicted by both the GAMs, while the BRT predicted a more continuous area of moderately high habitat suitability throughout the offshore region extending north to about 40°N (Figure 3e). All long‐beaked common dolphin models captured this species’ largely coastal distribution south of about 36°N, but the BRT showed an extension of low to moderate habitat suitability extending north along the entire coast (Figure 3b). The distribution pattern predicted by both GAMs is consistent with the documented occurrence of long‐beaked common dolphin in the study area, as central/southern California is considered the northern extent of this species’ normal range (Carretta et al., 2011; Gerrodette & Eguchi, 2011). There have been recent sightings of long‐beaked common dolphin north of 36°N (Ford, 2005; Huggins et al., 2011) and Ford (2005) anticipated that additional records of the species would occur during anomalously warm water periods. However, long‐beaked common dolphins are not sighted north of Pt. Conception consistently enough to validate the BRT predictions.

**FIGURE 4:**
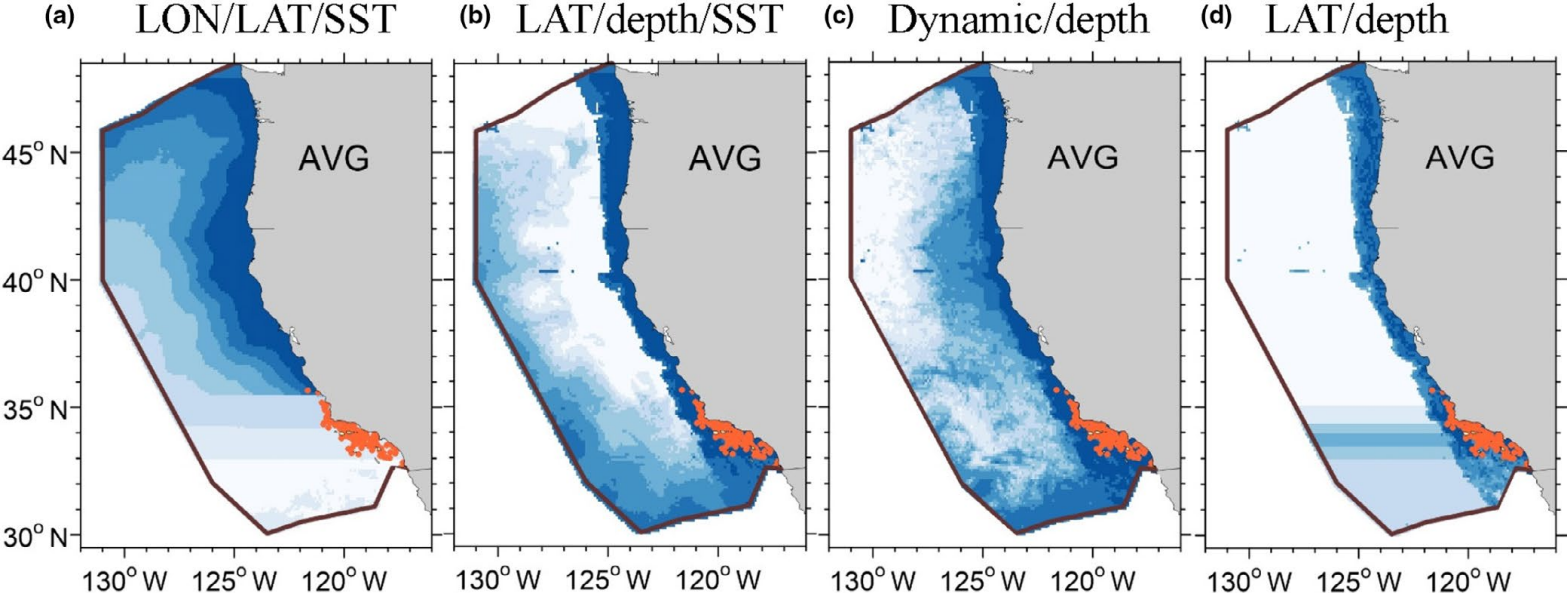
Example predictions of long‐beaked common dolphin habitat suitability from BRTs built with the 1991–2014 survey data and various combinations of predictor variables including (a) longitude (LON), latitude (LAT), and sea surface temperature (SST), (b) latitude, depth, and SST only, (c) dynamic and bathymetric variables only (SST, standard deviation of SST, mixed layer depth, sea surface height (SSH), standard deviation of SSH, and depth), and (d), a BRT developed using data south of 37°N that included latitude and depth only. Panels show the multiyear average (AVG) habitat suitability based on daily predictions covering the survey periods (summer/fall 1991–2014). Habitat suitability values are presented in eight equal‐numbered bins to better depict predicted distribution patterns. Predictions are shown for the study area (1,141,800‐km^2^). Orange dots show actual long‐beaked common dolphin sighting locations from the SWFSC 1991–2014 summer/fall ship surveys and illustrate the poor fit of these BRT predictions

During the model development phase, we attempted to improve the BRT prediction for long‐beaked common dolphin by building BRTs with various combinations of the dynamic, bathymetric, and spatial variables that could potentially eliminate the extension of low to moderate habitat suitability north of 36°N. Unfortunately, no combination of predictor variables we offered the BRT was able to successfully capture the limited distribution pattern of long‐beaked common dolphin in the southern inshore portion of the study area, and all model predictions were worse (Figure 4) than the original (Figure 3b). As an experiment, we also built BRTs using data only south of 37°N, but when making predictions on the entire study area, these models still predicted high habitat suitability in the northern portions of the study area and in some cases well offshore (Figure 4d). In addition, the inclusion of latitude created apparent modeling artifacts in some of the BRT predictions (Figure 4a,d).

The lower and upper 90% confidence intervals (CIs) of density/habitat suitability for the GAMs and BRTs showed overall similarities throughout the study area, with regional differences apparent in species distribution patterns for both the lower and upper CIs that were similar to differences apparent in the multiyear average density/habitat suitability comparisons (Figure 3). For the majority of the species, the upper CIs for the BRTs were higher across the study area than those of the GAMs (e.g., Figure 3b,c,e–g).

### 3.2 Predictive performance

The ability of the different model types to make accurate predictions during the novel 2014 year differed substantially, and based on the observed: predicted ratios, the density GAMs generally outperformed both the presence/absence GAMs and BRTs, particularly for northern right whale dolphin, fin whale, and humpback whale (Table 3). The density GAM had the best observed:predicted ratio (i.e., closest to 1) for six of the seven species, while the presence/absence GAM had the best ratio for northern right whale dolphin (Table 3).

**TABLE 3:** Summary of the final GAM and BRT models built with the 1991–2009 survey data, the number of sightings available for modeling (n), and their ability to accurately predict study area habitat suitability (HS)/density (Dens) for the novel year (2014)

Species	Predictor variables	Novel 2014 Obs:Pred
Short‐beaked common dolphin (*n* = 709)		
GAM HS	SST:LAT + SSH +year + MLD +SSTsd	1.05
BRT HS	LAT, SST, depth, MLD, SSH, SSTsd, SSHsd	1.89
GAM Dens	SST + SSH +depth + year +SSTsd	1.02*
Long‐beaked common dolphin (*n* = 122)		
GAM HS	LON:LAT + SSHsd	0.87*
BRT HS	LAT, depth, SST, SSH, MLD	0.40
GAM Dens	LON:LAT + SST	0.80
Northern right whale dolphin (*n* = 108)		
GAM HS	SST + depth	1.17
BRT HS	SST, MLD, LON, LAT, SSTsd, depth, SSH	0.88
GAM Dens	SST:LAT + depth	1.05*
Striped dolphin (*n* = 103)		
GAM HS	depth + SST	2.41
BRT HS	SSH, depth, LAT	2.46
GAM Den	depth + SST	2.10*
Risso's dolphin (*n* = 171)		
GAM HS	LON:LAT + MLD	0.52
BRT HS	LAT, depth, SST, LON, MLD, SSHsd, SSTsd. SSH	0.56
GAM Dens	LON:LAT + SST +depth	0.61*
Fin whale (*n* = 362)		
GAM HS	LON:LAT + year +SSH + SST	0.82
BRT HS	LAT, SSH, MLD, depth, SST, SSTsd, SSHsd	1.62
GAM Dens	SST:lat + year +SSH + depth +MLD	1.05*
Humpback whale (*n* = 292)		
GAM HS	SST + depth +SSHsd + SSH +year	2.87
BRT HS	SST, depth, MLD, SSTsd, SSHsd,SSH, year	1.78
GAM Dens	LON:LAT + year +depth + SSHsd	1.15*

Note

Variable abbreviations are as follows: depth, bathymetric depth; LAT, latitude; LON, longitude; MLD, mixed layer depth; SSH, sea surface height; SSHsd, standard deviation of SSH; SST, sea surface temperature; SSTsd, standard deviation of SST. The “LON:LAT” and “SST:LAT” terms in the GAMs indicate an interaction term. All models for short‐beaked common dolphin, fin whale, and humpback whale were also offered a year covariate to capture their change in abundance during the 1991–2009 survey years (see text for details). The novel 2014 Observed:Predicted (Obs:Pred) ratios reflect total study area values. For each species, the model with the best predictive performance (i.e., closest to 1) is marked with an asterisk (*).

**FIGURE 5:**
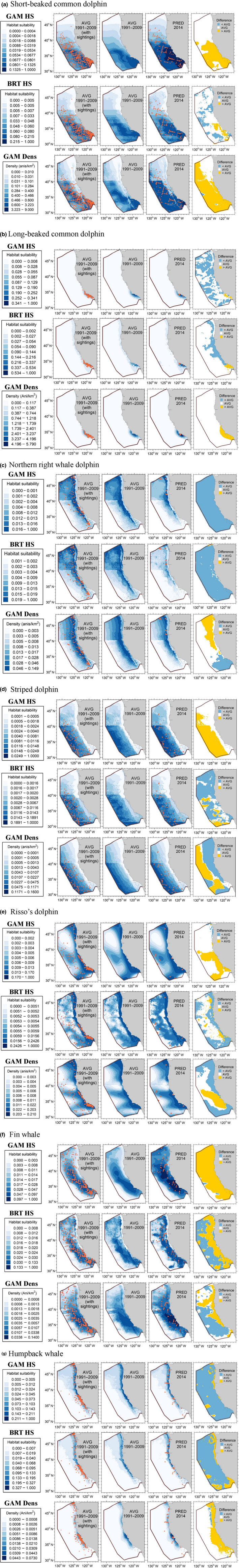
Predicted habitat suitability/density values from the 1991–2009 models compared to novel 2014 summer/fall predictions for (a) short‐beaked common dolphin, (b) long‐beaked common dolphin (c) striped dolphin, (d), northern right whale dolphin, (e) Risso's dolphin, (f) fin whale, and (g) humpback whale. Panels show the multiyear average (AVG) habitat suitability (HS)/density (Dens) values based on daily predictions covering the survey periods for summer/fall (July–December, 1991–2009). To enable comparisons among the different model types, habitat suitability/density values are presented in eight equal‐numbered bins based on the range of values for the 1991–2009 period with a 9th color added to emphasize the higher 2014 predictions. Predictions are shown for the study area (1,141,800‐km^2^). Orange dots show actual sighting locations from the summer/fall 1991–2009 and 2014 ship surveys, respectively. The difference between the predicted 2014 and 1991–2009 average habitat probability/density values (i.e., 2014 predictions minus 1991–2009 average predictions) are shown in the fourth panel. Blue represents predicted 2014 values that were lower than the 1991–2009 average (i.e., <0), white represents values that were similar to the 1991–2009 average (i.e., within a small density difference up to 0.01, depending on species and based on the range of absolute density values), and yellow represents values that were substantially higher than the 1991–2009 average

The plots of predicted 2014 distribution patterns as compared to the 2014 survey observations showed that both types of GAM were better able to predict shifts in distribution during the anomalously warm conditions in 2014 as compared to the BRTs (Figure 5). For example, two warm temperate/tropical species in our study, short‐beaked common and striped dolphins, have continuous distributions southward into Mexican waters (Mangels & Gerrodette, 1994; Perrin, Scott, Walker, & Cass, 1985), and the distribution of both species expanded to the north during the warm 2014 conditions, increasing their abundance throughout the CCE study area (Barlow, 2016; Becker et al., 2018). The density GAM was able to capture the northward expansion of both short‐beaked common and striped dolphins (i.e., swaths of higher‐than‐average density were predicted north of 40°N; Figure 5a,d). The presence/absence GAM was able to capture this northward shift for striped dolphin, while the BRTs predicted average to lower‐than‐average habitat suitability north of 40°N for both species (Figure 5a,d). The 2014 study area abundance estimate for short‐beaked common dolphin was almost twice as high as in previous years. For waters off Oregon and Washington, average abundance was more than five times higher than in previous years (Barlow, 2016) due to the northward expansion in distribution during the warm 2014 conditions. Both types of GAMs for short‐beaked common dolphin were able to capture the absolute increase in abundance/habitat suitability, while the BRT predicted average to lower‐than‐average habitat suitability throughout most of the study area (Table 3 and Figure 5a).

The long‐beaked common dolphin BRT predicted suitable habitat extending north along the coast of the entire study area in 2014, but lower than what was predicted for previous years. The 2014 predictions from both types of GAMs better matched the known distribution of this species in the southern nearshore region of the study area, and areas with the highest predicted density were consistent with long‐beaked common dolphin sighting locations during the 2014 survey (Figure 5b).

Among all the BRTs, the best observed:predicted habitat suitability ratio for the novel 2014 year was for northern right whale dolphin (0.88; Table 3). Interestingly, the difference plot for this species revealed that the BRT predicted lower‐than‐average habitat suitability for the majority of the study area, with a very small region near the northern border predicted to have higher‐than‐average habitat suitability for northern right whale dolphin (Figure 5c). During the 2014 survey, all sightings of northern right whale dolphin were north of 40°N, different from previous surveys where this species was sighted as far south as the Southern California Bight (Figure 5c). The density GAM also captured this northward shift in distribution in 2014, but the difference plot for the GAM contrasted sharply with that of the BRT, with higher‐than‐average density predicted for the northern portion of the study area and lower‐than‐average density predicted for the south (Figure 5c). For this species, the habitat suitability GAM had the worst observed:predicted ratio of the three models (1.17), and the difference plot was very similar to the BRT, with lower‐than‐average northern right whale dolphin habitat suitability predicted for the entire study area (Figure 5c).

All three types of models for Risso's dolphin overestimated density/habitat suitability in the study area in 2014, with observed:predicted ratios ranging from 0.52 to 0.61 (Table 3). There were both similarities and differences between the novel 2014 prediction plots for the different model types, yet none appeared to fully capture observed distribution patterns of Risso's dolphin during this novel year (Figure 5e). The difference plots for the Risso's dolphin 2014 predictions also showed similarities and differences for the different model types, with the two GAMs capturing similar patterns of higher‐than‐average predictions while the BRT and density GAM captured similar patterns of lower‐than‐average predictions (Figure 5e).

Predicted distribution patterns from both fin whale GAMs were fairly consistent with sighting data from the 2014 surveys, but the BRT predicted higher‐than‐average habitat suitability in the southwest corner of the study area, where there were no sightings of this species in 2014 or during the previous 1991–2009 surveys (Figure 5f). The density GAM had an observed:predicted abundance ratio close to unity (1.05), while study area habitat suitability was overpredicted by the presence/absence GAM (0.82) and underpredicted by the BRT (1.62; Table 3).

The density GAM for humpback whale captured the increase in the number of individuals in the study area in 2014 (Barlow, 2016; Becker et al., 2018), with higher‐than‐average predictions for the region between approximately 34°N and 38°N where there were multiple sightings during the 2014 survey (Figure 5g). Conversely, the presence/absence GAM and BRT predicted lower‐than‐average habitat suitability for this region in 2014 (Figure 5g), and the observed:predicted ratios for these two models show that they substantially underestimated habitat suitability for humpback whales in 2014 (Table 3).

## 4 DISCUSSION

GAMs and BRTs have been established as two commonly used modeling frameworks to guide spatial management and conservation strategies for cetaceans (e.g., Abrahms et al., 2019; Gilles et al., 2016; Hazen et al., 2017; Redfern et al., 2019). Improving the application of SDMs for spatial planning and conservation efforts requires a better understanding of the strengths and weaknesses of these modeling methods. Both methods are used to model nonlinear covariate responses, but the mechanics of the two approaches differ, as GAMs use flexible smoothing functions while BRTs use binary splits (regression trees). Our study compared both the explanatory power (i.e., model goodness of fit) and predictive power (i.e., performance on a novel dataset) of habitat suitability GAMs and BRTs, as well as density GAMs, for a taxonomically diverse suite of cetacean species using a robust set of systematic survey data. Below we provide details on the models’ performance and discuss species‐specific characteristics that could have affected these results.

### 4.1 Model comparison: explanatory versus predictive performance

The key environmental variables (i.e., those that had the most influence on the respective model) and general trend of their response curves (Figure S1) were similar in the final 1991–2014 GAMs and BRTs, as were the study area ratios of observed: predicted density/habitat suitability and the overall distribution patterns for the majority of species (Table 1, Figure 3). These results are similar to those of Scales et al. (2016), who found the ranking of variable performance, model response curves, and spatial predictions of GAMs, BRTs, and RFs similar when predicting the foraging habitats of gray‐headed albatross (*Thalassarche chrysostoma*). The percentage of explained deviance, AUC, and TSS metrics were consistently higher for the BRTs (Table 1), suggesting that this type of model has higher explanatory ability than the GAMs; however, the predictive power of the BRTs was lower than both types of GAMs based on both the ratios of observed‐to‐predicted values for the novel 2014 year (Table 3) as well as the spatial plots of predicted values to the 2014 actual survey sightings (Figure 5).

This result is consistent with the findings of Oppel et al. (2012), who found that machine‐learning techniques (BRT, RF, and Maxent) showed excellent explanatory performance when discriminating between presence/absence, but poor performance when predicting on independent test data. They found a similar pattern when they used RF to develop density models, and attributed the inferior predictive performance to machine‐learning techniques overfitting more than parametric models (Oppel et al., 2012). In our case, it is likely that overfitting in the BRTs made it more difficult to predict on the anomalous 2014 oceanic conditions that were not reflected in the training datasets, whereas the smooths in the GAMs were better able to handle such differences. Specifically, GAMs extend the splines between predictor and variable partial response to predict on novel conditions while BRTs assume a static relationship when predicting out of bounds (Zurell, Elith, & Schröder, 2012). However, with any modeling framework, when extrapolating outside the range of values used to build the models, results should be interpreted cautiously, particularly if data are not available for cross‐validation (Becker et al., 2014; Mannocci, Roberts, Miller, & Halpin, 2017).

Year was included in both types of GAMs for short‐beaked common dolphin, fin whale, and humpback whale, while the BRT only included year for humpback whale (i.e., year had lower relative variable contribution than the random variable in both the short‐beaked common dolphin and fin whale models). Thus, the GAMs appeared to capture absolute changes in population size as populations recovered (fin and humpback whales) or moved into the CCE (short‐beaked common dolphin).

One of the advantages of BRTs is the implicit incorporation of interaction terms, such as between latitude and longitude, which must be explicitly defined in a GAM. However, for many of our BRTs, latitude and/or longitude created odd modeling artifacts in the prediction surfaces. For example, a spatial interaction term (latitude:longitude) was included in both types of GAM for long‐beaked common dolphin, and these models accurately captured this species’ limited distribution in the study area (Figure 3b). However, when latitude and/or longitude was included in the long‐beaked common dolphin BRT, these models produced ecologically unreasonable swaths of habitat suitability along latitude lines (e.g., Figure 4a,d). Further, latitude did not capture the expected patterns of long‐beaked common dolphin habitat in the final 1991–2014 BRT, because low‐to‐moderate habitat suitability was predicted for areas along the entire U.S. west coast (Figure 3b), outside the normal range of this species (Carretta et al., 2011; Gerrodette & Eguchi, 2011). Spatial terms were effective in the BRTs for some of the species considered here (e.g., striped dolphin, Figure 3d), so we suggest that modelers use care when including spatial terms in BRTs.

### 4.2 Model comparison: species with widespread vs. limited distribution

During summer and fall, short‐beaked common dolphins and fin whales are known to occur throughout large portions of our study area (Barlow, 2016; Barlow & Forney, 2007; Becker et al., 2016; Calambokidis et al., 2015). Both types of the 1991–2014 GAMs and the BRTs successfully captured the distribution patterns of short‐beaked common dolphins and fin whales in the study area (Table 2, Figure 3a,f), suggesting that both model types have strong explanatory capability for species with widespread distributions. Predictive performance differed by model type for these species, as the GAMs predicted study area abundance for the novel year within 5% (density GAMs) to 18% (presence/absence GAMs) of what was observed, while the BRTs underpredicted habitat suitability by more than 60% for fin whales and almost 90% for short‐beaked common dolphins (Table 3).

In contrast, humpback whales and long‐beaked common dolphins have more limited coastal distributions in our study area, with the latter typically occurring south of about 36°N (Barlow, 2016; Barlow & Forney, 2007; Becker et al., 2016; Calambokidis et al., 2015; Carretta et al., 2011). Elith et al. (2008) suggested that one of the advantages of BRTs over GAMs is that they could handle sharp discontinuities when modeling species with distributions that occupied only a small proportion of the sampled environmental space. Our results are inconsistent with this finding, as the humpback whale and long‐beaked common dolphin density GAMs performed well for both species, the presence/absence GAM had the best predictive performance among the three model types for long‐beaked common dolphin yet the worst for humpback whale, and the BRTs for both these species exhibited poor predictive ability (Table 3). The BRT 1991–2014 spatial predictions for both species also had issues as evident from the habitat suitability plots; the long‐beaked common dolphin BRT showed suitable habitat north of the typical range for this species (Figure 3b), and the humpback whale BRT showed low‐to‐moderate habitat suitability in areas to the northwest and well offshore, where there have been no sightings of this species during the SWFSC surveys (Figure 3g). Surprisingly, the BRTs for both species had some of the highest explained deviance, AUC, and TSS values among all the models (Table 1). This illustrates that both threshold‐independent (AUC) and threshold‐dependent (TSS) measures can be misleading in cases when species “prevalence,” that is, the proportion of the study area in which a species occurs, is low (Fiedler et al., 2018; Fourcade, Besnard, & Secondi, 2018; Somodi, Lepesi, & Botta‐Dukát, 2017). Our results are consistent with others who have suggested that AUC alone is not a robust measure of SDM predictive performance because it does not provide information on the spatial distribution of model errors (Lobo, Jiménez‐Valverde, & Real, 2007) and that model selection based solely on TSS can be misleading (Ruete & Leynaud, 2015). This result has important implications for management efforts in areas where the distribution of a species is poorly known, because reliance on the BRT AUC and TSS metrics alone could result in misguided conservation strategies (e.g., ill‐defined boundaries for protected areas, ineffective mitigation measures, etc.).

### 4.3 Model comparison: species occurring in more versus less heterogeneous habitats

The northern right whale dolphin is a cool‐temperate species that occurs primarily in slope and shelf waters in the study area, which are oceanographically dynamic. Northern right whale dolphins exhibit southward distribution shifts into the Southern California Bight during cool‐water periods, such as the winter months (Becker et al., 2014; Dohl, Norris, Guess, Bryant, & Honig, 1980; Forney & Barlow, 1998). The striped dolphin is a tropical species inhabiting warm offshore waters of the study area (Barlow, 2016; Becker et al., 2016; Forney et al., 2012), which are oceanographically less dynamic than the shelf and slope waters of the California Current Ecosystem (Chelton, Bernal, & McGowan, 1982; Haury, 1976; Hickey, 1979). Becker et al. (2010) found that the complexity of a species’ habitat influenced the predictive ability of GAMs and that greater sample sizes were required to parameterize models for species that inhabit more heterogeneous or dynamic environments.

The 1991–2014 spatial distribution patterns of northern right whale dolphins and striped dolphins were successfully captured by all three models (Table 2, Figure 3c,d), suggesting that both model types have strong explanatory capability for species inhabiting habitats of varying complexity. However, the novel predictions for 2014 underestimated striped dolphin abundance (density GAM) and habitat suitability (presence/absence GAM and BRT) by over a factor of two (Table 3). The range of striped dolphin extends continuously from the study area south to waters offshore Mexico (Mangels & Gerrodette, 1994; Perrin et al., 1985). During the anomalously warm water conditions in 2014, the available striped dolphin habitat within the study area likely increased substantially, resulting in an unprecedented influx of animals into the study area that was not fully captured by any model type. However, both types of GAM captured the northward distribution shift that was not evident in the BRT novel model predictions (Figure 5d).

The northern right whale dolphin GAMs and BRT all demonstrated good performance when making 2014 predictions, and among the BRT models for all species, the predictions for this species were the most accurate (observed:predicted habitat suitability ratio = 0.88; Table 3). During the unusually warm year 2014, the distribution of northern right whale dolphins shifted into the northern portions of the study area, and there were no sightings south of 40°N where sightings had been common during the cooler years 1991–2009 (Figure 3c). Both of the 1991–2009 GAMs for northern right whale dolphin were able to predict this northward shift in 2014, with zero to low densities predicted in the south and highest densities in the north (Figure 5c). The 1991–2009 BRT also captured this shift, as evident from the BRT 2014 predictions that show areas of highest habitat suitability in the northeast corner of the study area (Figure 5c).

The difference plots (Figure 5c) for the three models were dissimilar; however, as the density GAM predicted higher‐than‐average density in the north and lower‐than‐average density in the south. In contrast, the presence/absence GAM and the BRT predicted lower habitat suitability for almost the entire study area. In this case, both habitat suitability models erroneously implied a lower study area abundance of northern right whale dolphins, while the observed survey results indicated that there were nearly twice as many animals within a smaller (northern) area (Barlow, 2016). The density GAM successfully predicted this observed increase in abundance during 2014 (observed:predicted density ratio of 1.05, Table 3). This result could be attributed in part to the different response variables, since the measure of presence/absence is not affected by the number of animals, whereas it has a direct impact on density. This result emphasizes the fact that an apparent decrease in habitat suitability does not necessarily equate to a decrease in abundance within a study area and thus has important considerations when using presence/absence predictions in a management context (Boyd et al., 2018). In a future study, we plan to develop methods for using machine‐learning techniques such as BRTs to predict density from these multidecadal survey data and associated detection factors.

The anomalously warm conditions in 2014 provided a unique opportunity to assess the predictive ability of the models given the substantial shifts in distribution exhibited by many of the species considered here, including both striped and northern right whale dolphins. Our study area represents the northern range of striped dolphin (Mangels & Gerrodette, 1994; Perrin et al., 1985), and in 2014, there was likely an influx of animals into the study area from Mexican waters. Conversely, the distribution of northern right whale dolphins shifted into the northern portions of the study area in 2014, likely expanding their distribution north of the CCE study area into Canadian waters. Such movement of animals into or outside of a politically defined study area can present challenges to marine spatial planners focused on developing study area specific conservation measures, particularly when geographically limited data make it difficult to discern between apparent changes in abundance versus shifts in distribution. Future efforts to develop SDMs based on study areas defined by a species range could help inform management decisions and lead to a greater understanding of species ecology.

With the exception of the striped dolphin presence/absence GAM built with the 1991–2014 survey data, all the models for both striped and northern right whale dolphins show an abrupt discontinuity that runs east to west at 40°N (Figures 3c,d and 5c,d). This reflects the location of the Mendocino Escarpment, a bathymetric feature evident in many of the models that included depth as a significant predictor, although the abrupt change in habitat suitability/density was most striking in the plots for striped and northern right whale dolphins. Although the Mendocino Escarpment is quite deep, empirical evidence suggests that this deep‐water feature does have manifestations in terms of surface marine life (e.g., Pyle, 2005) and thus may provide an ecological component relevant to habitat modeling. Increased biodiversity in this pelagic region may be due to the offshore transport of upwelling filaments associated with Cape Mendocino (Keister & Strub, 2008). Conversely, studies of submarine canyons and seamounts suggest that these types of deep bathymetric features may provide hotspots for surface marine life and that enhanced dynamics such as increased vertical nutrient fluxes and material retention can promote productivity and subsequently attract higher trophic levels (Morato, Hoyle, Allain, & Nicol, 2010; Santora, Zeno, Dorman, & Sydeman, 2018). Internal waves interacting with the Mendocino Escarpment can create deep‐ocean mixing (Althaus, Kunze, & Sanford, 2003; Di Lorenzo et al., 2006), although more definitive studies are needed to see if there are mechanistic explanations for biological surface effects.

### 4.4 Model comparison: a species for which previous GAMs have been challenging

Previous GAMs developed for Risso's dolphin using subsets of the data used here did not perform as well as expected, and there was poor correlation between predicted density patterns and sighting data used to build the models (Becker et al., 2010; Forney et al., 2012). Sighting data reveal a longitudinal hiatus in the distribution of Risso's dolphins within the study area, with sightings concentrated either along the continental shelf (mainly south of 38°N) or in offshore deep waters (Barlow, 2016; Barlow & Forney, 2007). Becker et al. (2016) suggested that this sighting pattern might represent two separate populations of Risso's dolphin and included an interaction term between the 200‐m isobaths and latitude to capture the observed spatial distribution in a GAM. For this study, we wanted to compare the ability of the GAMs and BRT to capture the distribution pattern of Risso's dolphin without including this interaction term and using additional sighting data from the 2014 survey.

Similar to previous studies, the multiyear average density plot produced by the 1991–2014 density GAM did not correlate well with the sighting data, as there were no sightings in high‐density regions and multiple sightings in the lowest density regions (Figure 3e). The presence/absence GAM exhibited similar patterns to the density GAM and also failed to accurately capture the observed 1991–2014 distribution patterns. The 1991–2014 BRT average habitat suitability plot did a better job at capturing the disjunctive longitudinal sighting pattern south of 40°N but also showed highest habitat suitability continuous along the coast from approximately 38°N to 42°N and in the southeast corner of the study area where there were few sightings during the 1991–2014 surveys (Figure 3e). The fact that none of the three models were able to capture the distribution patterns of Risso's dolphin likely indicates that the environmental variables offered to the models are not effective proxies for their habitat and prey. Large and small squid account for approximately 85% of the diet of Risso's dolphin (Pauly, Trites, Capuli, & Christensen, 1998). Squid are typically found at depths >200 m (Childress & Seibel, 1998), and identifying an available proxy that better captures the ecological processes driving squid distribution may improve the explanatory power of Risso's dolphin SDMs. Given their poor explanatory performance, it is not surprising that both the GAMs and BRT for Risso's dolphin also had poor predictive performance (Table 3).

## 5 CONCLUSIONS

This study provided a unique opportunity to compare the performance of two commonly used SDM modeling frameworks, GAMs and BRTs, for a diverse suite of cetacean species to better understand strengths and limitations of each approach. All three models (density GAMs and presence/absence GAMs and BRTs) exhibited good explanatory performance and did well at predicting spatial patterns for species that have widespread distributions throughout the study area and for species that inhabit oceanographically diverse (i.e., more or less dynamic) environments. For species with limited distributions in our study area, the BRTs were not able to accurately capture their spatial distribution patterns despite strong performance as indicated by commonly used model evaluation metrics, confirming previous studies that have suggested that both AUC and TSS can be misleading when used to evaluate SDMs (Fiedler et al., 2018; Lobo et al., 2007; Ruete & Leynaud, 2015). Further, the inclusion of latitude and longitude in some of the BRTs, most notably for long‐beaked common dolphin, resulted in odd modeling artifacts and predicted spatial distribution patterns opposite to those documented for this species.

When predicting on anomalous novel data, the density GAMs exhibited higher predictive performance than the presence/absence GAMs and substantially higher predictive performance than the BRTs. This is likely due to both the different response variables and the different fitting algorithms. Since the density GAMs are predicting absolute abundance, they are better able to respond to changes in the number of animals present in the study area, particularly for species whose distributions shrink but abundance increases (i.e., northern right whale dolphin in 2014). Similar to previous studies (Leathwick et al., 2006; Oppel et al., 2012), we found that BRTs had good explanatory power for most species but were not able to make accurate predictions on novel data, likely due to overfitting. Perhaps a better method of model selection to avoid overfitting could improve the predictive power of BRT models.

While there may be no single best modeling framework for predicting cetacean density or presence/absence, our results have provided an improved understanding of some of the strengths and limitations of both GAMs and BRTs. These findings support the use of both model types for describing species relationships, but suggest that a cautionary approach should be used when applying BRTs to anomalous novel data, and when including spatial terms (latitude, longitude) in the suite of potential predictors. Model ensembles have been shown to be a powerful tool for leveraging the weaknesses and strengths of different model types (Abrahms et al., 2019; Woodman et al., 2019) and may be a useful option for future species distribution modeling work. Continual efforts to evaluate and improve the predictive performance of species distribution models will aid in the conservation and management of cetacean species worldwide.

## CONFLICT OF INTEREST

None declared.

## AUTHOR CONTRIBUTION

**Elizabeth A. Becker:** Conceptualization (equal); Data curation (equal); Formal analysis (lead); Investigation (lead); Methodology (equal); Writing‐original draft (lead); Writing‐review & editing (lead). **James V. Carretta:** Conceptualization (equal); Formal analysis (supporting); Investigation (supporting); Methodology (supporting); Writing‐original draft (supporting); Writing‐review & editing (supporting). **Karin A. Forney:** Conceptualization (equal); Data curation (equal); Formal analysis (supporting); Investigation (supporting); Methodology (equal); Writing‐original draft (supporting); Writing‐review & editing (supporting). **Jay Barlow:** Data curation (equal); Formal analysis (supporting); Funding acquisition (equal); Investigation (supporting); Methodology (supporting); Writing‐original draft (supporting); Writing‐review & editing (supporting). **Stephanie Brodie:** Formal analysis (supporting); Methodology (supporting); Writing‐original draft (supporting); Writing‐review & editing (supporting). **Ryan Hoopes:** Investigation (supporting); Writing‐original draft (supporting); Writing‐review & editing (supporting). **Michael G. Jacox:** Data curation (equal); Formal analysis (supporting); Writing‐original draft (supporting); Writing‐review & editing (supporting). **Sara M. Maxwell:** Funding acquisition (equal); Project administration (supporting); Writing‐original draft (supporting); Writing‐review & editing (supporting). **Jessica V. Redfern:** Conceptualization (supporting); Methodology (supporting); Writing‐original draft (supporting); Writing‐review & editing (supporting). **Nicholas B. Sisson:** Formal analysis (supporting); Writing‐original draft (supporting); Writing‐review & editing (supporting). **Heather Welch:** Formal analysis (supporting); Methodology (supporting); Writing‐original draft (supporting); Writing‐review & editing (supporting). **Elliott L. Hazen:** Funding acquisition (equal); Methodology (supporting); Project administration (supporting); Writing‐original draft (supporting); Writing‐review & editing (supporting). 

## Supporting information

Figure S1

## Data Availability

The cetacean data used in this study are publicly available on the Cetacean and Sound Mapping website (https://cetsound.noaa.gov/cda). The ROMS output is publicly available on the U.C. Santa Cruz Ocean Modeling and Data Assimilation website (https://www.cencoos.org/data/models/roms/westcoast).
