# Supplementary material for: Performance evaluation of cetacean species distribution models developed using generalized additive models and boosted regression trees
Source: Ecol Evol. 2020 May 11;10(12):5759–84. doi: 10.1002/ece3.6316 (PMC7319248; doi:10.1002/ece3.6316)

**Figure S1**. Final SDM response curves for (a) short-beaked common dolphin, (b) long-beaked common dolphin, (c) striped dolphin, (d), northern right whale dolphin, (e) Risso’s dolphin, (f) sperm whale, (g) fin whale, and (h) humpback whale. All models were offered the same suite of environmental covariates that included: SST = sea surface temperature, MLD = mixed layer depth, SSH = sea surface height, sdSSH = standard deviation of SSH, sdSST = standard deviation of SST, depth = bathymetric depth, LAT = latitude, and LON = longitude. Models for short-beaked common dolphin, fin whale, and humpback whale were also offered a year term (see text for details). For both the habitat suitability and density GAMs, the degrees of freedom for nonlinear fits are shown in the parentheses on the y-axis and the shading reflects 2x standard error bands (i.e., 95% confidence interval). For the habitat suitability BRTs, the percentages shown on the x-axis reflect the contribution of each variable to explained deviance.


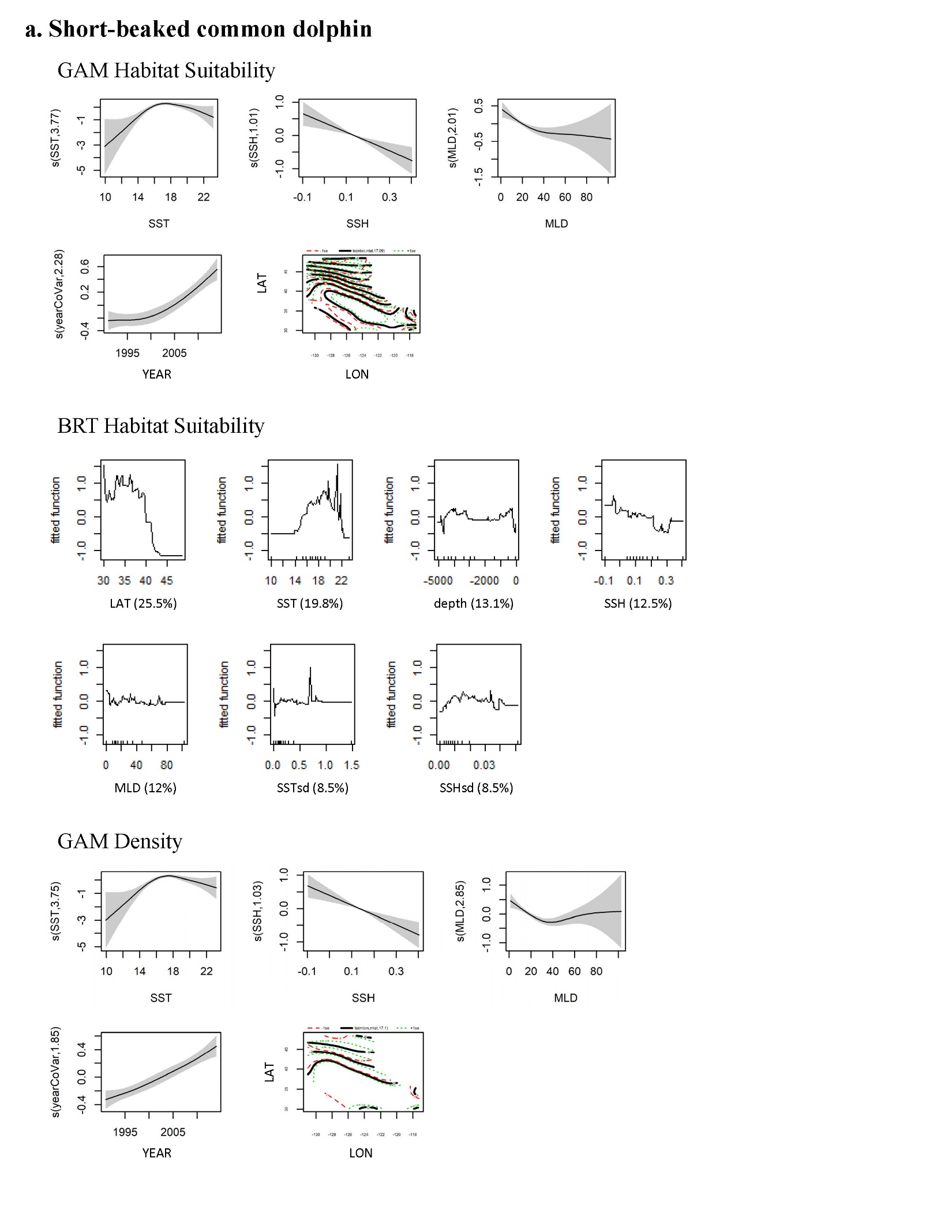


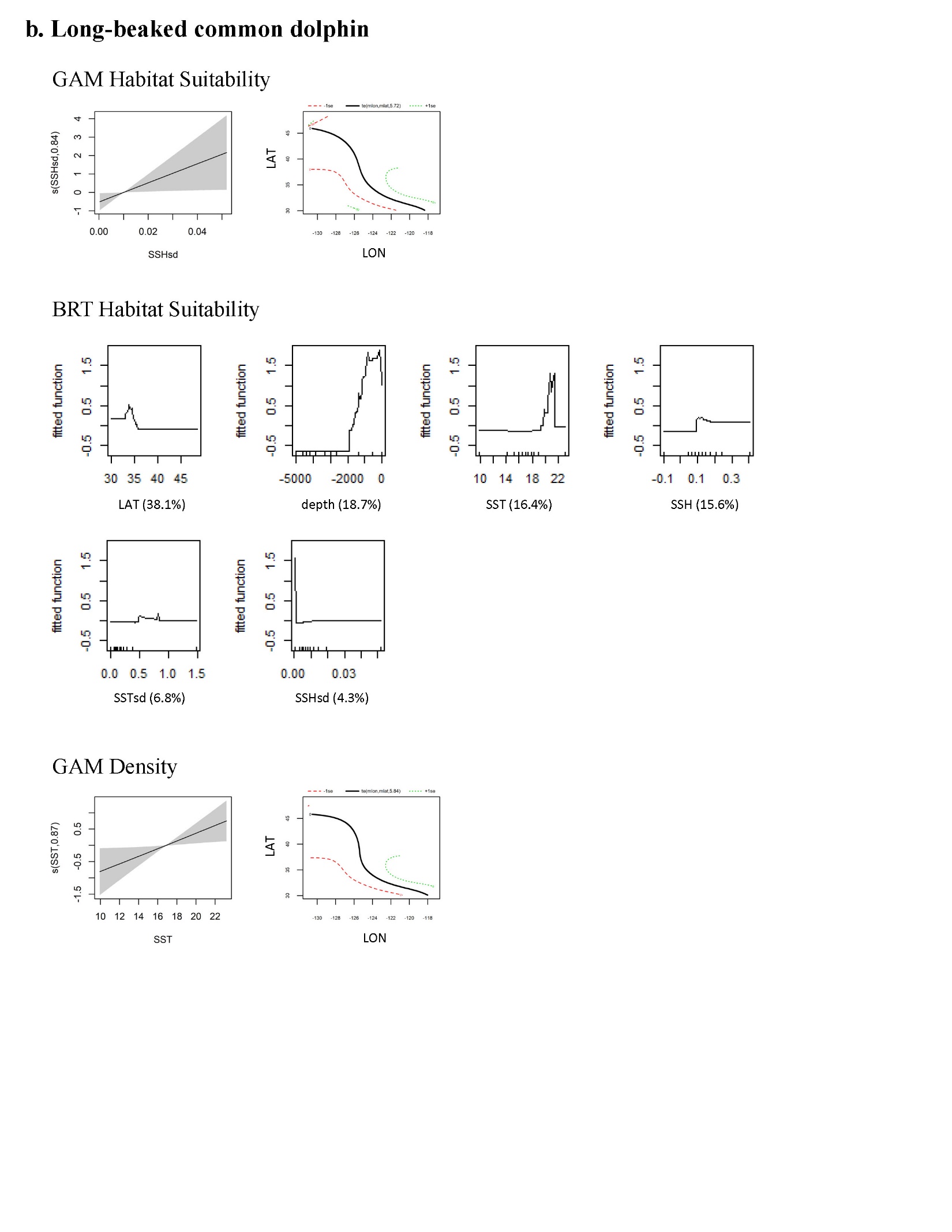


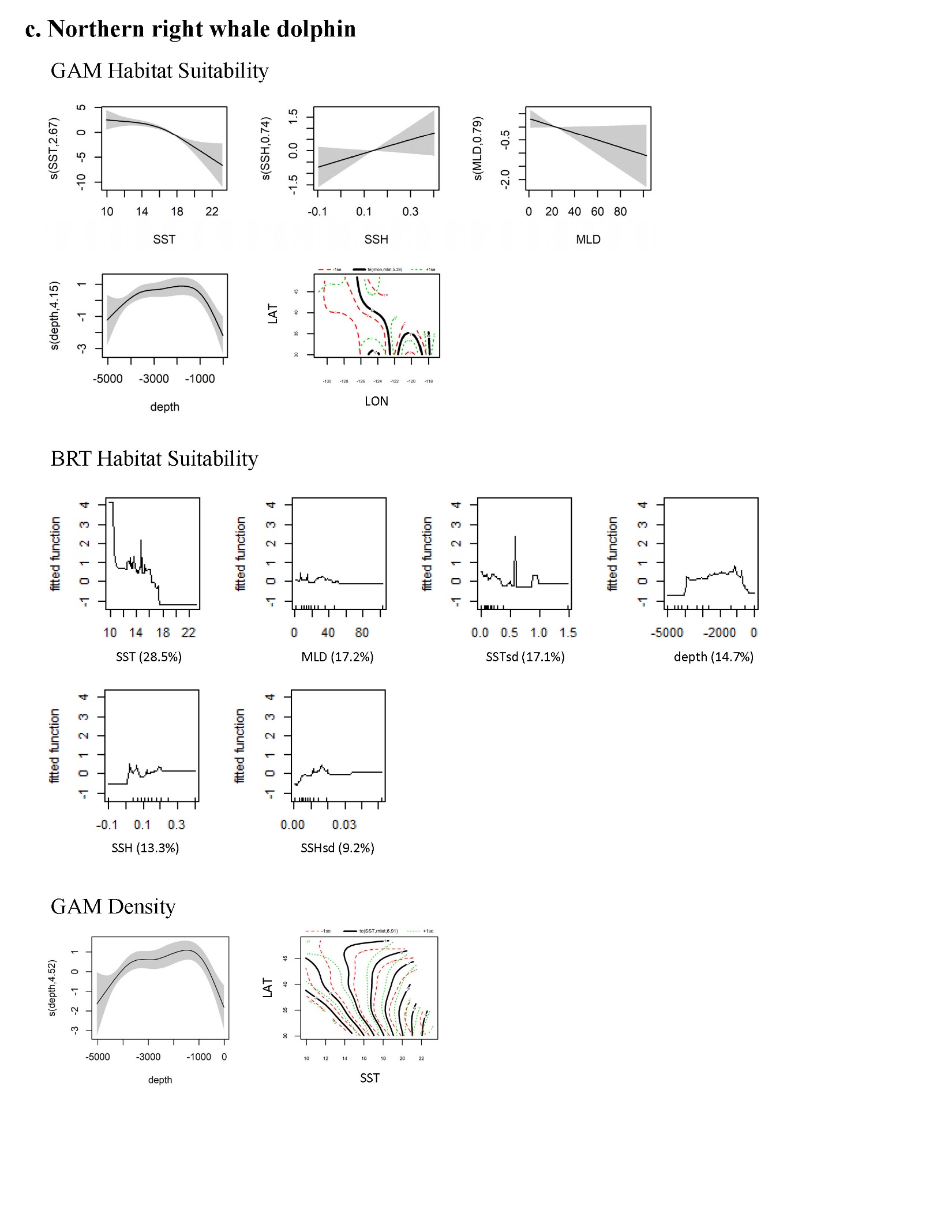


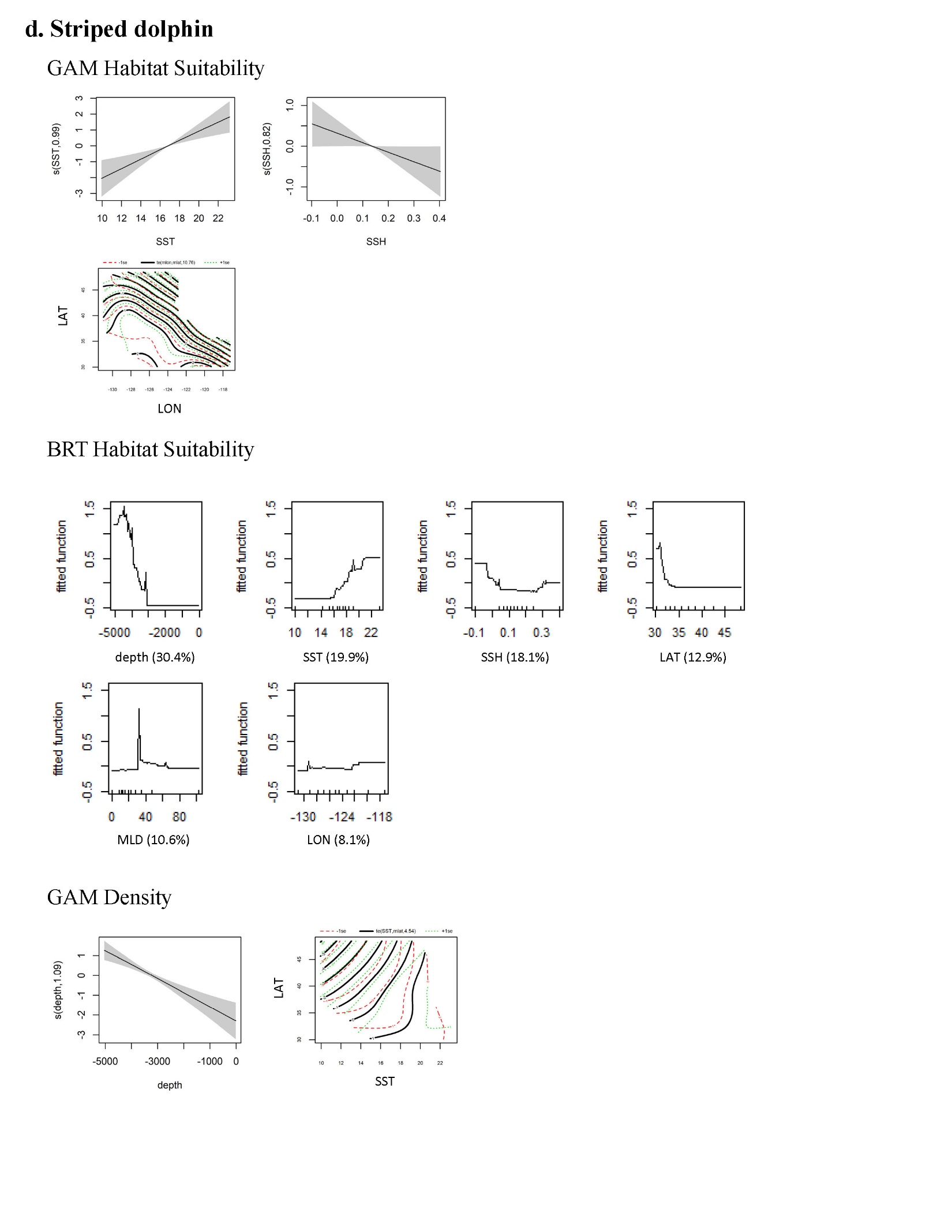


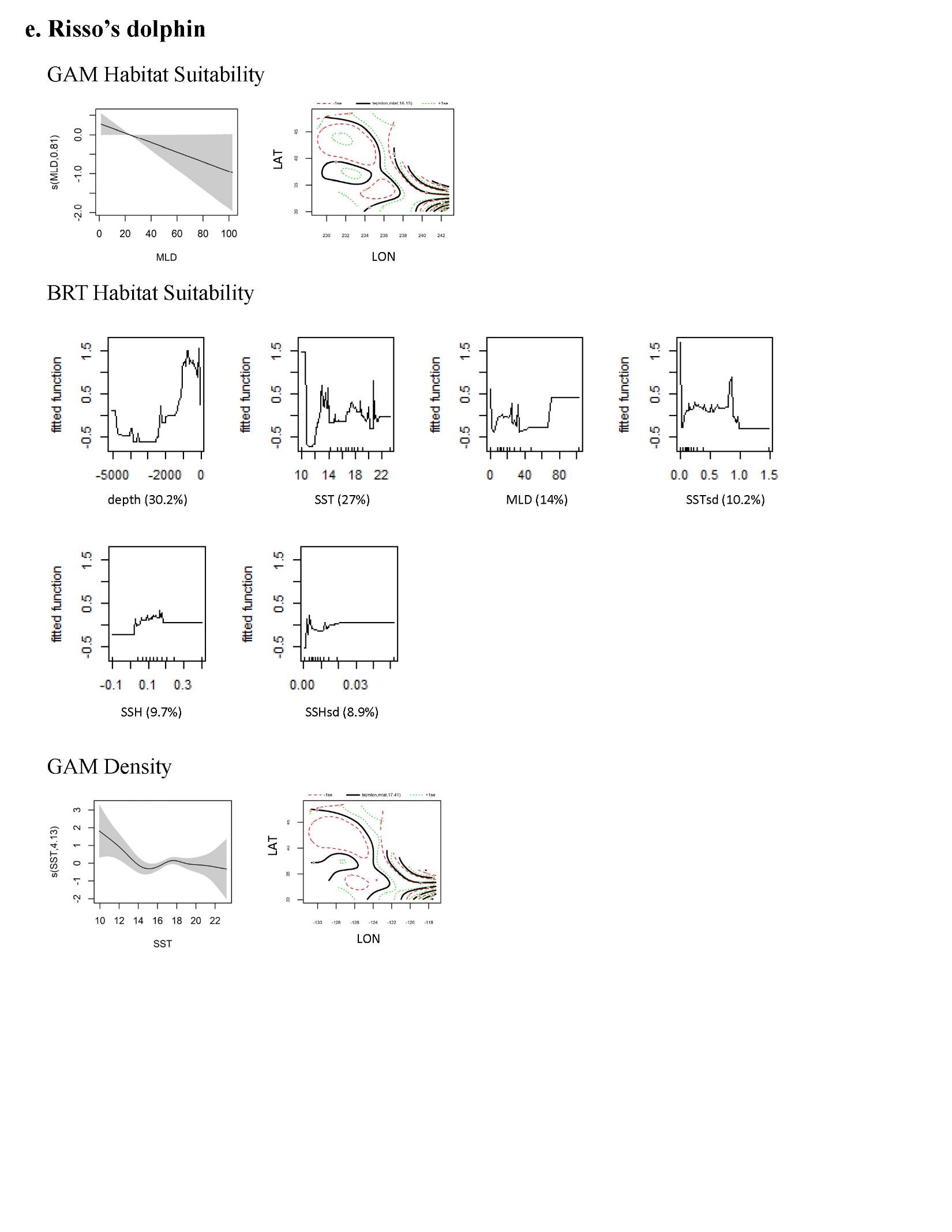


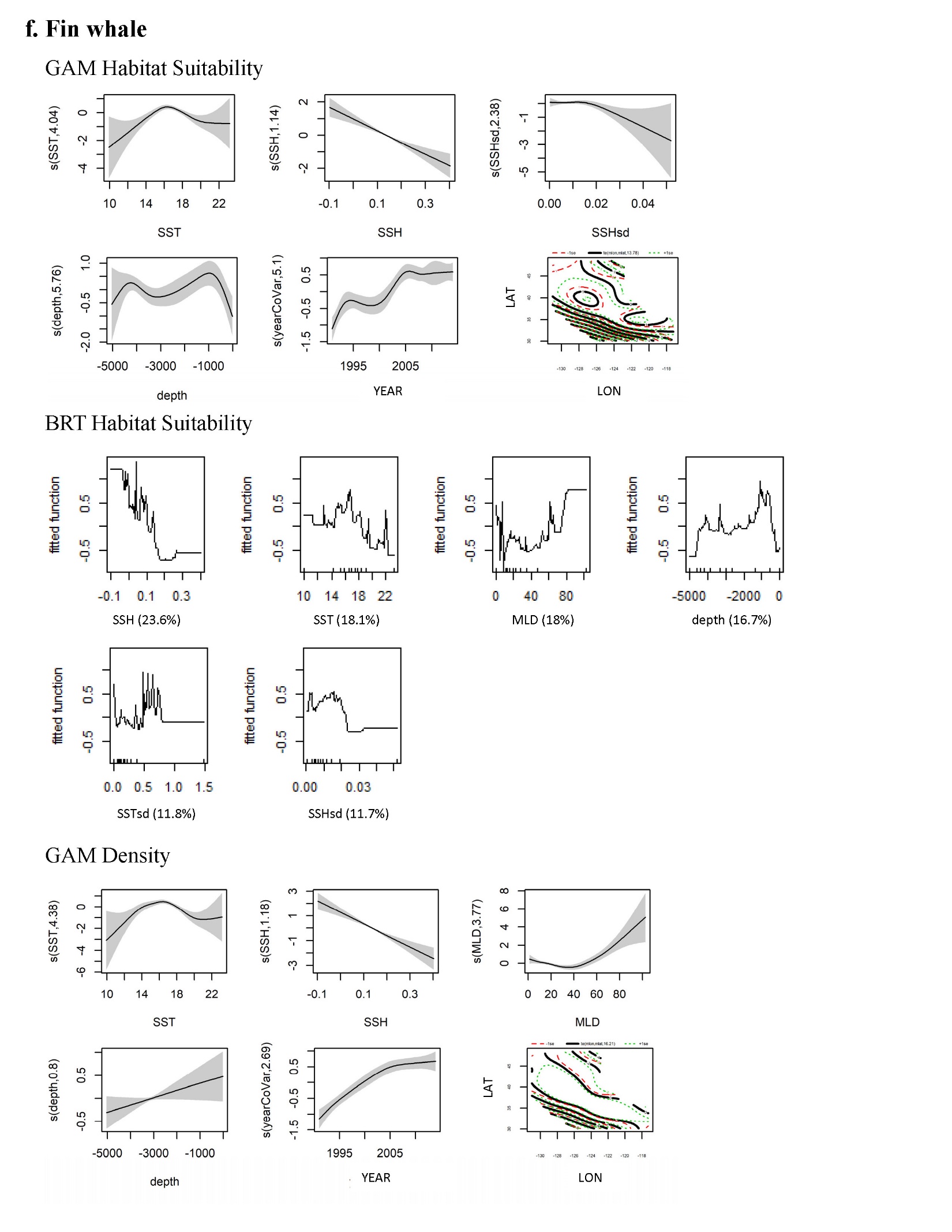


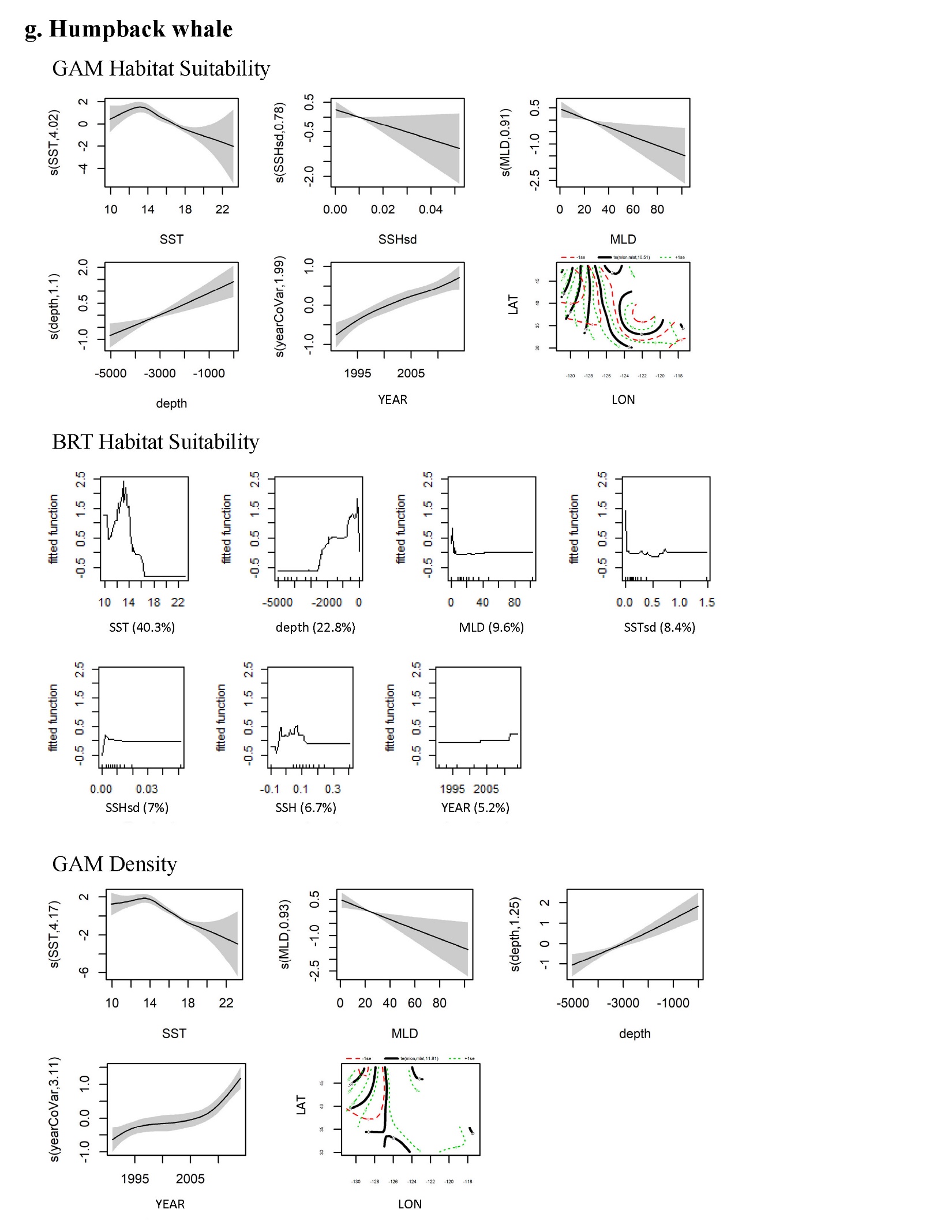

Supplement: Supplementary file 1 — Figure S1 [file ECE3-10-5759-s001.docx]
